# Predicting satisfaction with vocational status among people with serious mental illness in vocational services: the role of perceived skills and support

**DOI:** 10.3389/fpsyt.2025.1533227

**Published:** 2025-02-26

**Authors:** Gili Hoter Ishay, Gil Goldzweig, Ilanit Hasson-Ohayon, Marc Gelkopf, Efrat Shadmi, David Roe

**Affiliations:** ^1^ The Occupational Therapy Department, Ono Academic College, Kiryat Ono, Israel; ^2^ Department of Community Mental Health, Faculty of Social Welfare and Health Sciences, University of Haifa, Haifa, Israel; ^3^ School of Behavioral Sciences, The Academic College of Tel Aviv Yaffo, Tel Aviv, Israel; ^4^ Department of Psychology, Bar Ilan University, Ramat Gan, Israel; ^5^ The Cheryl Spencer Nursing Department of Nursing, Faculty of Social Welfare and Health Sciences, University of Haifa, Haifa, Israel

**Keywords:** supported employment, individual placement and support, job satisfaction, vocational satisfaction, employability

## Abstract

**Background:**

This study aimed to explore the role of perceived skills and support in satisfaction with vocational status to better address the vocational needs and recovery goals of individuals with serious mental illness (SMI). It focuses on three service types: individual placement and support (IPS), sheltered workshops, and vocational support centers.

**Method:**

The study is a cross-sectional analysis of the nationwide Israeli PR-PROM project data. A sample of 2,968 participants diagnosed with SMI and their service providers self-reported their perceived skills, support, and satisfaction with vocational status.

**Results:**

We found perceived skills and support to be primary predictors of vocational satisfaction across all groups. There were significant differences in satisfaction with vocational status among the three service types: IPS users reported higher satisfaction than sheltered workshop and vocational support center users. The service-provider evaluations reflected similar trends, with higher satisfaction ratings for more integrative services. Additionally, discrepancies were observed between participant and provider satisfaction ratings, varying by vocational service type.

**Discussion:**

The study highlights the importance of perceived skills and support in enhancing satisfaction with vocational status for people with SMI regardless of the vocational service type. Whereas objective factors like employment status and salary contribute to vocational rehabilitation and quality of life, subjective perceptions of skills and support play a significant role in satisfaction with vocational status. The results suggest that vocational rehabilitation services should monitor consumers’ perceptions of skills and support to improve their vocational satisfaction, which can promote personal well-being and social integration.

## 1 Introduction

Employment status and vocational functioning have been recognized as key elements in the recovery of people with serious mental illness (SMI). Accordingly, employment is a common desirable recovery goal positively associated with a range of life domains, including symptomatology, subjective measures of recovery, and satisfaction (1, 2). Research has indicated that individuals with and without disabilities who perceive themselves as trained and having adequate vocational skills and support are more likely to achieve and maintain employment and show higher psychosocial functioning, well-being, and job satisfaction (3, 4). Furthermore, perceived skills and support might influence an individual’s motivation to seek employment opportunities and their resilience in facing workplace challenges.

Job satisfaction plays a crucial role in overall well-being and serves as a significant predictor of employee engagement and organizational commitment (5). In today’s dynamic labor market, where job tenure continues to decline (6), the importance of job satisfaction has gained even more prominence. This trend is further exacerbated by increasing demand for re-skilling and employee commitment to lifelong learning. Research has indicated that the perception of opportunities for skill acquisition is intricately linked to the level of organizational support provided to employees, which directly affects their job satisfaction (7, 8). When organizations actively foster an environment conducive to learning and development, employees are more likely to feel valued and satisfied in their roles. Jackson and Wilton (9) found that individuals who feel confident in their vocational skills often experience a heightened sense of job security. This sense of security benefits their overall job performance and contributes to enhanced health and well-being. Thus, fostering job satisfaction through skill development and organizational support emerges as a pivotal strategy for enhancing employee health, performance, and loyalty in the workplace.

Most individuals with SMI express a desire to work (10, 11) but face high unemployment rates (20%–55%), indicating a need for supportive interventions (10, 12) to overcome common individual, social, and system barriers (13). These barriers often include low self-perception, motivation, cognitive deficits, and fears related to their illness and past experiences. They can hinder their job search and retention but do not universally hinder employability (11).

In addition, a lack of work experience and effective job-seeking strategies often limit the employability of people with SMI (14). Social stigma is another barrier to job acquisition and retention. In particular, people with SMI may confront negative experiences that reduce their perceived work-related competence, enhance self-stigma, and affect perceived job discrimination, nonworking role models, and unconstructive opinions from coworkers (15, 16). These perceptions emphasize the importance of vocational rehabilitation services providing skills training and promoting competence perceptions. It has long been recognized that people with SMI, like people with physical disabilities, have the need and right to acquire the necessary skills and environmental support to facilitate their vocational aspirations and recovery (17).

Despite research showing that people with SMI have significantly lower rates of satisfaction with work and vocational status than their typical counterparts, the topic has not received much scholarly attention (18, 19). Historically, research has focused mostly on objective vocational outcomes, such as employment rates, income, and the use of vocational rehabilitation services (20); less attention has been given to subjective work aspects, such as satisfaction with vocational status and job security (20, 21). In recent years, consensus on the benefits of employment to recovery has slowly shifted toward more complex consideration of individual experiences, like personal meaning, contextual factors, and their role in the recovery journey (22, 23). *Employment* is perceived as a pathway to meaningful engagement and citizenship. These essential aspects of recovery (24) indicate that individuals can find meaning in activities beyond paid work. Consumer perspectives underscore the value of social connections and a sense of identity, broadening the more conservative benefits to recovery (22). Objective factors, such as employment status, job stability, workplace accommodations, and salary, as well as subjective experiences, including personal fulfillment, enjoyable experiences, and alignment of job tasks with personal interests or values (19, 22, 25), influence the definition of job satisfaction. The term *job satisfaction* had been investigated mainly in individual placement and support (IPS) services because it refers to competitive employment. However, in our study, it is used in its broadest sense, referring to satisfaction with vocational status in all services investigated.

The most effective model for vocational integration of people with SMI has been IPS (26, 27). The IPS considers the need for community integration and competitive employment and has consistently demonstrated higher vocational outcome rates than traditional vocational rehabilitation (28–30). Importantly, people in IPS who secured jobs aligned with their preferences reported higher job satisfaction and longer tenure (31, 32).

Alongside IPS in the form of *supported employment*, some people with SMI use traditional prevocational services focused on employability and skills acquisition as part of their vocational rehabilitation. Israel, for example, provides two types of prevocational services: *sheltered vocational workshops*, which require competitive employment-like demands and focus on productivity and outputs, and *vocational support centers*, which focus more on skills training and leisure-like activities. Neither involves formal employer–employee relationships or salaries because they are *pre*vocational rehabilitation services. Instead, these prevocational services promote skill acquisition and environmental support to facilitate vocational aspirations as part of the process toward job attainment (17).

Because vocational rehabilitation services providers are considered part of the support offered in these services, their perceptions may act as facilitators or barriers to consumers’ successful job acquisition and satisfaction (19, 33). However, the accumulating research has revealed gaps between stakeholders’ ratings on a range of treatment-related issues (34, 35), emphasizing the methodological value of assessing multiple perspectives rather than relying solely on self-reported or service-provider ratings. For example, service providers’ beliefs in their clients’ work ability directly affect service efficacy and client satisfaction (19). Moreover, research findings have suggested that providers tend to rate consumer outcomes in vocational rehabilitation services conservatively, often linking more integrative services to better outcomes (33).

The primary significant benefits of IPS over prevocational services are its vocational outcomes, such as employment rate and job tenure. However, nonvocational, subjective outcomes like quality of life or job satisfaction did not show benefit from IPS (27, 36). Moreover, job satisfaction does not appear as a vocational or nonvocational outcome of IPS in most studies (27). One study that examined social firms found that people with higher occupational self-efficacy—and who received workplace accommodations and greater social support—were more likely to experience greater job satisfaction (31).

Considering the notable importance of skills, support, and job satisfaction, the purpose of the present study was to investigate the relationship between perceived skills/support and satisfaction with vocational status among people with SMI who consumed IPS-based *supported employment* compared to two prevocational services offered to people with SMI in Israel (*vocational support centers* and *sheltered workshops*). The first hypothesis was that perceived social support and perceived vocational skills would relate to satisfaction with vocational status among people consuming the three vocational services, over and beyond the effect of psychiatric symptoms. Because satisfaction with vocational status has rarely been examined in either type of vocational service (prevocational and supported employment), we hypothesized that the results would show differences in satisfaction with vocational status. Last, we hypothesized that there would be differences between the providers’ and the participants’ ratings of satisfaction with vocational status.

## 2 Materials and methods

### 2.1 Design and participants

Data were collected as part of the Israeli Psychiatric Rehabilitation Patient Reported Outcome Measurement project (PR-PROM), which included an annual routine assessment of multidimensional measures delivered nationally for all psychiatric rehabilitation clients in Israel (see (37) for further details on the research project). The Ministry of Health’s Helsinki Committee approved the study, and written informed consent was obtained after a detailed description of the project was provided to the participants.

This study was a cross-sectional analysis of 2,968 participants (21% of the PR-PROM sample) who had consumed a vocational service for at least 10 months during the year preceding the data collection. All participants had a psychiatric disability of at least 40% (determined by a medical committee that included a psychiatrist), according to the National Security Institute in Israel and case records of SMI. Specifically, all had been diagnosed with a severe mental disorder according to the *Diagnostic and Statistical Manual of Mental Disorders* (5th ed.) criteria (38). The participants’ mean age was 46.16 years (*SD* = 12.26; range 20.00–85.00);^1^ 1,674 (56%) identified as men. 397 (13%) were married or living with a partner, and 1,709 (62%) had 12 or more years of education. **Table 1** shows the sample’s full demographic characteristics by service. Significant between-service differences were found in age, gender, education, and hospitalization.

**Table 1:** Sociodemographic and background data by vocational rehabilitation service (*N* = 2,968).

Demographic	(1) Vocational support center (*N *= 295)	(2) Sheltered workshop (*N *= 1868)	(3) Supported employment (*N *= 805)	Total (*N *= 2,968)	Difference
Age (yr) *M* ± *SD* (range) Age categories *n* (%): 17–25 yr 26–45 yr 46–65 yr 66 yr and older	49.39 ± 12.40 (21–76) 21 (3.2) 208 (32.0) 365 (56.0) 577 (6.0)	47.33 ± 12.37 (20–85) 119 (4) 1,250 (40) 1,543 (49) 218 (7)	41.98 ± 10.84 (20–72) 80 (6) 803 (59) 464 (34) 19 (1)	46.16 ± 12.26 (20–85) 220 (4) 261 (44) 2,372 (46) 299 (6)	*F*(2,2900) = 68.72, *p* < 0.0001 1 vs. 2 *p* < 0.029* 1 vs. 3 *p* < 0.0001** 2 vs. 3 *p* < 0.0001**
Gender: Men *n* (%)	156 (53.0)	1018 (54.5)	500 (62.0)	1,674 (56)	χ^2^(2) = 14.92, *p* < 0.0001 1 vs. 2 ns 1 vs. 3 p < 0.006 3 vs. 2 p < 0.0001
Marital status *n* (%) Married or living with a partner	39 (13)	224 (12)	134 (17)	397 (13)	χ^2^(2) = 10.84, *p* < 0.004 1 vs. 2 ns 1 vs. 3 ns 3 vs. 2 ns
Ethnicity *n* (%) Jewish Arab	255 (91) 25 (9)	1,637 (93) 130 (7)	1,225 (91) 123 (9)	702 (93) 54 (7)	χ^2^(2) = 1.012, *p* < 0.63 *ns*
Birth country *n* (%) Israel Eastern Europe Other/unknown	201 (70.5) 55 (19.0) 32 (11.0)	1,242 (68) 353 (19) 229 (13)	570 (73) 110 (14) 100 (13)	2,013 (70.0) 518 (18.0) 361 (12.5)	χ^2^(2) = 11.25, *p* < 0.025 1 vs. 2 ns 1 vs. 3 ns 3 vs. 2 p < 0.005
Education *n* (%) ≤ 12 yr > 12 yr	209 (77) 64 (23)	1,119 (64) 619 (36)	381 (65) 373 (37)	1,709 (62) 1,056 (38	χ^2^(2) = 70.67, *p* < 0.0001 1 vs. 2 p < 0.0001 1 vs. 3 p < 0.0001 3 vs. 2 p < 0.0001
Number hospitalization days last 10 yr *M* ± *SD* (range) Hospitalization days >0 *n* (%)	98.56 ± 234.80 (0–2,287) 146 (50)	109.64 ± 231.67 (0–2,364) 1,035 (57)	64.39 ± 154.0 348 (44)	96.29 ± 214.65 (0–2,364) 1,529 (53)	*F*(2,2899) = 12.31, *p *< 0.0001 1 vs. 2 p < 1.00 ns 1 vs. 3 p < 0.06 ns 2 vs. 3 p < 0.0001**

*ns*, not statistically significant. *p <.05; **p <.01.

### 2.2 Vocational services

Participants were recruited from three types of vocational services in Israel: two forms of “prevocational” employment services—*vocational support centers* and *sheltered workshops*—and supported employment. *Vocational support centers* aim to provide a productive occupation in a flexible, noncompetitive environment adjusted to the person’s current abilities. They place little emphasis on output or efficiency. The centers offer manufacturing tasks (an exclusively manufactured product for which the people with SMI are fully responsible for planning, producing, marketing, and branding) or occupational (e.g., self-advocacy, interview skills, meeting schedules, and interpersonal communication) and cognitive (e.g., problem-solving, decision-making, and time management) skills-training interventions with fewer demands than sheltered workshops.

*Sheltered workshops* provide an adjusted working environment, typically manufacturing-like, without employer–employee relationships. Here, consumers develop their working habits and vocational skills. The work structure is flexible within the activity duration (4–7 hours per day), difficulty level, and job type. Each sheltered service provides 30 to 100 consumers with “rehabilitation incentives” according to their actual outputs. The demands on the individual tend to increase over time, enabling internal ranking, promotion, and skills development. Each workshop has a rehabilitation service provider (a professional mental health rehabilitation worker, such as a social worker, psychologist, or occupational therapist) who accompanies the consumers’ rehabilitation process.

*Supported employment (IPS)* is a support service aimed at enabling the successful and meaningful integration of people with psychiatric disabilities into the open, competitive employment market as soon as possible. It is the most integrative vocational service, characterized by a “natural” competitive work environment. The work environment varies according to the occupational field and type of work. A vocational consultant supports the consumers throughout the process of finding a full- or part-time competitive job and successfully keeping the job. The well-established IPS model and its principles have inspired supported employment services in Israel.

### 2.3 Instruments

The participants completed the Patient Reported Outcome Measures (PROMs). The PROMs was created based on existing assessment tools and additional tools developed specifically for the national project (PR-PROM). In addition to administering the PROMs to each participant, the SMI respondents’ service providers completed identical questionnaires from their points of view. Sociodemographic data and data on salaries and the number of days/hours spent consuming vocational services were based on self-reports. All questionnaires were tested in a previous pilot project and showed satisfying reliability and validity (37).

#### 2.3.1 Satisfaction with vocational status

Satisfaction with vocational status was assessed using a single item on the PROMs that originated from the Manchester Short Assessment of Quality of Life (39): “How satisfied are you with your vocational status?” The item was scored on a five-point Likert-type scale from 1 (*not satisfied at all*) to 5 (*very much satisfied*). The service provider assessed the participant’s satisfaction regarding their vocational status using a similar item.

#### 2.3.2 Perceived effect of symptoms

The perceived effect of symptoms on vocational functioning was assessed by a single item from the Sheehan Disability Scale (40): “In the last month, to what extent have the symptoms disrupted your work/school activities?” Respondents scored the item on a five-point Likert scale from 1 (*not at all*) to 5 (*very much*).

#### 2.3.3 Perceived skills and support

Consumers completed single items regarding perceived skills and perceived support, respectively, “To what extent do you feel you have capabilities and skills that can help you succeed in your vocational status?” and “To what extent do you feel you have the help and support to succeed in developing your vocational status?” Both items were scored on a five-point Likert-type scale from 1 (*not at all*) to 5 (*very much*). These items were adopted from the Maryland Assessment of Recovery in People with Serious Mental Illness Scale (41).

### 2.4 Data analysis

Descriptive statistics and comparisons of background variables between participants in the three vocational service categories were performed using univariate analyses, including *post hoc* comparisons. Hierarchical multiple linear regression models were performed to predict satisfaction with vocational status by background variable and to assess perceived skills and support for each vocational service using consumer and provider assessments.

## 3 Results

### 3.1 Comparing work-related data and satisfaction with vocational status by vocational service

**Table 2** describes the sample’s work-related data by vocational service. Most (about 60% of each service) participants worked 5 days a week; 83% reported working more than 4 hours daily. Most of the participants in the vocational support (94%) and sheltered workshop (90%) groups reported receiving one-tenth of an average salary because there are no working relationships and often no salaries involved in these types of services. In contrast, only 26% of the supported employment group reported receiving one-tenth of an average salary; most reported higher salaries.

**Table 2:** Comparison of Work-Related Data by Vocational Rehabilitation Service.

Variable	(1) Vocational support center group (*n* = 295)	(2) Sheltered workshop group (*n* = 1,868)	(3) Supported employment group (*n* = 805)	Total (*N *= 2,968)	Difference
**Workdays/week: n (%)** **1–4** **5–6**	108 (38.0) 178 (62.0)	700 (38.5) 1,116 (61.5)	276 (35.0) 506 (65.0)	1,084 (38.0) 1,800 (62.0)	χ^2^(2) = 2.47, *p *< 0.29 *ns*
**Monthly payment: n (%)** **≤ NIS 1,200** **> NIS 1,200**	256 (96.0) 10 (4.0)	1,540 (89.5) 180 (10.5)	189 (26.0) 726 (74.0)	1,985 (74.0) 720 (27.0)	χ^2^(2) = 1,117.36 *p *< 0.0001 1 vs. 2 *p *< 0.0001 1 vs. 3 *p *< 0.0001 2 vs. 3 *p *< 0.0001
**Work hours/day: n (%)** **1–3** **≥ 4**	102 (35.0) 188 (65.0)	251 (14.0) 1,588 (86.0)	135 (17.0) 659 (83.0)	488 (17.0) 2,435 (83.0)	χ^2^(2) = 83.51 *p* < 0.0001 1 vs. 2 *p *<0.0001** 1 vs. 3 *p *<0.0001** 2 vs. 3 *p *<0.0001**
**Satisfaction with vocational status (consumer): *M* (*SD*)**	3.670 (1.125)	3.580 (1.180)	3.710 (1.090)	3.620 (1.150)	*F*(2, 2396) = 3.67,*p *< 0.025 1 vs. 2 *p* < 0.62 *ns* 1 vs. 3 *p* < 1 *ns* 2 vs.3 *p* < 0.027*
**Satisfaction with vocational status (service provider): *M* (*SD*)**	3.245 (0.910)	3.510 (0.910)	3.830 (0.820)	3.570 (0.910)	*F*(2, 2728) = 53.37,*p* < 0.0001 1 vs. 2 *p* <0.0001** 1 vs. 3 *p* <0.0001** 2 vs.3 *p* <0.0001**

Note. ns = not statistically significant. *p <.05; **p < .01.

**Table 2** reports data concerning satisfaction with vocational status levels. The mean scores for satisfaction with vocational status reported by consumers differed significantly between the three groups. *Post hoc* comparisons revealed that satisfaction in the supported employment group was significantly higher than in the sheltered workshop group (Bonferroni *p* < 0.027: vocational support center *M* = 3.670, *SD* = 1.125; sheltered workshop *M* = 3.580, *SD* = 1.180; supported employment *M* = 3.710, *SD* = 1.090), *F*(2, 2936) = 3.70, *p* < 0.025).

Service provider reports showed a trend similar to that of the participants: Mean satisfaction with vocational status significantly differed between the groups, and *post hoc* comparisons showed that all comparisons of vocational services significantly differed (Bonferroni *p* < 0.0001: vocational support center *M* = 3.245, *SD* = 0.920; sheltered workshop *M* = 3.510, *SD* = 0.915; supported employment *M* = 3.830, *SD* = 0.820), *F*(2, 2728) = 53.37, *p* < 0.0001).

### 3.2 Comparing satisfaction with vocational status between providers and consumers

The vocational support center service group of consumers reported a significantly higher level of satisfaction with vocational status than the providers reported (consumer *M* = 3.67; provider *M* = 3.24), *t*(262) = 5.26, *p* < 0.0001. However, providers in the supported employment group reported significantly higher levels of satisfaction with vocational status than consumers did (consumer *M* = 3.71; provider *M* = 3.83), *t*(757) = 3.52, *p* < 0.0001.

### 3.3 Predicting satisfaction with vocational status: consumers

**Table 3** summarizes the hierarchical regressions predicting self-reported satisfaction with vocational status. The predictors included sociodemographic variables, impact of symptoms, perceived vocational skills, and perceived support. In the vocational support service group, less symptom interference with work predicted higher satisfaction with vocational status. In the sheltered workshop group, being older, being male, and having less symptom interference significantly predicted higher satisfaction with vocational status. In the supported employment group, higher salaries significantly predicted higher satisfaction with vocational status. For all groups, lower education significantly predicted higher satisfaction with vocational status. Further, across all three groups, the largest significant contribution to the prediction model was the subjective vocational resources of perceived vocational skills and perceived support.

**Table 3:** Hierarchical regression analysis: summary of predictors of satisfaction with vocational status (self-reported).

Variable	Vocational support center group					Sheltered workshop group					Supported employment				
	B	*SE*	β	*t*	*p* <	B	*SE*	β	*t*	*p* <	B	*SE*	β	*t*	*p* <
Block 1: Sociodemographic															
Age	0.01	0.01	0.07	0.86	0.3909 *ns*	0.01	0.00	0.06	2.06	0.0393*	-0.002	0.004	-0.02	-0.38	0.7017 *ns*
Sex (0=male;1=female)	-0.06	0.17	-0.02	-0.33	0.7411 *ns*	0.15	0.06	0.06	2.31	0.0212*	0.06	0.10	0.03	0.62	0.5377 *ns*
Marital status (0=single; 1=married)	-0.11	0.24	-0.03	-0.45	0.6547 *ns*	-0.14	0.10	-0.04	-1.45	0.1476 *ns*	-0.005	0.12	-0.002	-0.04	0.9701 *ns*
Ethnicity (0=Jew, 1=Arab)	-0.03	0.30	-0.01	-0.09	0.9277 *ns*	-0.14	0.12	-0.03	-1.14	0.2529 *ns*	-0.17	0.18	-0.04	-0.96	0.3376 *ns*
Birth country (Israel *vs*. other)	-0.02	0.20	-0.01	-0.10	0.9242 *ns*	-0.03	0.07	-0.01	-0.40	0.6928 *ns*	0.00	0.11	0.00	0.03	0.9729 *ns*
Education (0=< 12 yr; 1=< 12 yr)	-0.55	0.21	-0.20	-2.70	0.0075**	-0.24	0.07	-0.10	-3.49	0.0005**	-0.28	0.09	-0.13	-3.02	0.0026**
	***F*(6, 188) = 1.74, *p* < 0.205 *ns*, *R* ^2^Δ = 0.044, total adjusted *R* ^2^ = 0.013**					*F*(6, 1307) = 3.96, *p* < 0.001**, *R* ^2^Δ = 0.018, total adjusted *R* ^2^ = 0.013					***F*(6, 548) = 1.64, *p* < 0.135 *ns*, *R* ^2^Δ = 0.018, total adjusted *R* ^2^ = 0.007**				
Block 2: Illness-related															
Hospitalization days (past 10 years)	0.00	0.00	0.07	0.93	0.3556 *ns*	0.00	0.00	0.04	1.38	0.1687	-0.02	0.10	-0.01	-0.18	0.8542 *ns*
Symptom interference with work (higher score = more interference)	-0.12	0.06	-0.14	-2.00	0.0467*	-0.14	0.02	-0.17	-6.03	0.0001**	0.02	0.19	0.01	0.11	0.9151 *ns*
	***F*(2, 186) = 2.63, *p* < 0.075 *ns*, *R* ^2^Δ = 0.026, total adjusted *R* ^2^ = 0.030**					*F*(2, 1305) = 19.14, *p* < 0.0001**, *R* ^2^Δ = 0.028, total adjusted *R* ^2^ = 0.040					*F*(2, 546) = 11.97, *p* < 0.0001**, *R* ^2^Δ = 0.041, total adjusted *R* ^2^ = 0.045				
Block 3: Work-related															
Workdays/week (0 = 1–4; 1 = 5–6)	-0.72	0.52	-0.32	-1.36	0.1740 *ns*	0.16	0.13	0.06	1.22	0.2221 *ns*	-0.02	0.10	-0.01	-0.18	0.8542 *ns*
Work hours/day (0 = 1–3; 1 = 4+)	1.14	0.58	0.47	1.97	0.0498 *ns*	0.11	0.17	0.03	0.67	0.5055 *ns*	0.02	0.19	0.01	0.11	0.9151 *ns*
Salary (0 = <NIS 1200; 1 = < NIS 1200)	0.30	0.45	0.05	0.66	0.5091 *ns*	0.11	0.11	0.03	1.01	0.3114 *ns*	0.38	0.11	0.15	3.31	0.001**
	***F*(3, 183) = 2.57, *p* < 0.056 *ns*, *R* ^2^Δ = 0.038, total adjusted *R* ^2^ = 0.054**					*F*(3, 1302) = 4.36, *p* < 0.005**, ***R* ^2^Δ = 0.009, total adjusted *R* ^2^ = 0.047**					*F*(3, 543) = 4.14, *p* < 0.006**, ***R* ^2^Δ = 0.021, total adjusted *R* ^2^ = 0.061**				
Block 4: External and internal resources															
Perceived vocational skills	0.20	0.08	0.19	2.52	0.0125*	0.22	0.03	0.21	7.48	0.0001**	0.22	0.03	0.21	7.48	0.0001**
Perceived support in vocational success	0.37	0.08	0.36	4.71	0.0001**	0.27	0.03	0.28	10.03	0.0001**	0.27	0.03	0.28	10.03	0.0001**
	***F*(2, 181) = 29.36, *p* < 0.0001, *R* ^2^Δ = 0.219, total adjusted *R* ^2^ = 0.28**					*F*(2, 1300) = 132.58, *p* < 0.0001**, ***R* ^2^Δ = 0.16, total adjusted *R* ^2^ = 0.21**					*F*(2, 541) = 67.06, *p* < 0.0001**, ***R* ^2^Δ = 0.18, total adjusted *R* ^2^ = 0.245**				
**Total model**	*F*(13, 181) = 6.74, *p* < 0.0001**, adjusted *R* ^2^ = 0.28					*F*(13, 1300) = 27.45, *p* < 0.0001**, **adjusted *R* ^2^ = 0.21**					*F*(13, 542) = 14.83, *p* < 0.0001**, **adjusted *R* ^2^ = 0.245**				

SE, standard error; *ns*, not significant. *p <.05; **p <.01.

The bold values show the contribution of a specific set of predictors to explain the variance in satisfaction with vocational status.

### 3.4 Predicting satisfaction with vocational status: service providers

**Table 4** presents the contribution of the subjective vocational resources, perceived vocational skills, and perceived vocational support to satisfaction with vocational status as reported by service providers in the three vocational groups. Predictors in the model included sociodemographic variables, illness-related variables, and perceived skills and support. The block of perceived support in vocational success significantly contributed to satisfaction with vocational status, as reported by the providers in each group. However, perceived vocational skills were a significant predictor only for the sheltered workshop and supported employment groups.

**Table 4:** Contribution of Subjective vocational resources to prediction of satisfaction with vocational status (service provider report) by vocational rehabilitation service.

Group	b coefficient	SE	β	*t*	*p* <
Vocational support center: *F*(2, 163) = 3.20, *p* < 0.043, *R* ^2^Δ = 0.032; Total model: *F*(13, 163) = 2.66, *p* < 0.0001**, adjusted *R* ^2^ = 0.10**					
Perceived vocational skills	0.009	0.073	0.01	0.12	0.906 *ns*
Perceived support in vocational success	0.15	0.071	0.18	2.06	0.0415*
Sheltered workshop: *F*(2, 1214) = 26.58, *p* < 0.0001**, *R* ^2^Δ = 0.038; Total model: *F*(13, 1214) = 9.57, *p* < 0.0001**, adjusted *R* ^2^ = 0.11					
Perceived vocational skills	0.14	0.026	0.16	5.31	0.0001*
Perceived support in vocational success	0.055	0.024	0.071	2.35	0.0190*
Supported employment: *F*(2, 505) = 22.40, *p*< 0.0001, *R* ^2^Δ = 0.074; Total model: *F*(13, 505) = 7.17, *p* < 0.0001**, adjusted *R* ^2^ = 0.14					
Perceived vocational skills	0.11	0.04	0.13	2.76	0.006*
Perceived support in vocational success	0.17	0.039	0.20	4.35	0.0001**

SE, standard error; *ns*, not significant. *p <.05; **p <.01.

## 4 Discussion

This study’s primary goal was to examine the role of perceived skills and support predict satisfaction with vocational status among people with SMI consuming three forms of employment services: IPS (supported employment), sheltered workshops, and vocational support centers.

A comparison of variables predicting satisfaction within each group revealed a consistent pattern: among all three groups, the association between perceived skills/perceived support and satisfaction with vocational status was significant and higher than the association of any other sociodemographic or clinical variable with satisfaction with vocational status. Higher education levels and the perceived impact of symptoms on functioning predicted lower satisfaction with vocational status but much less than perceived skills and perceived support, which contributed approximately 20% to the variance in satisfaction with vocational status.

This finding emphasizes the importance of the perceptions of having skills and support to promoting satisfaction with vocational status for people with SMI, regardless of the type of support. Working in competitive employment, having a full-time job, and other objective work variables are frequently perceived as “most important” for the vocational rehabilitation and quality of life of people with SMI (29, 42). Indeed, the observed differences in satisfaction with vocational status between groups in our study, may be partially explained by the higher rates of competitive employment achieved in the SE group. However, our results indicate that more factors contribute to satisfaction with vocational status beyond work status or other objective work-related variables. These findings align with previous research that emphasized the importance of the skills and support (30) upon which most psychiatric rehabilitation services rely (43). These findings may also relate to the general workforce’s “skilling-up” process, which places higher importance on learning new skills and improving existing skills to increase job satisfaction, especially for younger employees (44).

Our results reveal a gap between providers and users in evaluating consumer satisfaction with vocational status. Providers evaluated the participants’ satisfaction with vocational status as highest for those in the most integrative service and lowest for those in the most intensive support option (vocational support center). However, consumers in the less integrative service reported higher satisfaction with vocational status than their providers. Thus, these findings reveal gaps between providers and consumers in evaluating satisfaction—and the direction of the gap varies according to the service. These findings align with past research showing that providers tend to downplay consumer satisfaction in less integrative and less competitive services, perhaps because it does not correspond with the ideology and desire for community integration (45). Our study’s findings should alert service providers who might be similarly blinded to the dissatisfaction of consumers who nevertheless achieved supported employment. Although their employment signifies greater independence, they may downplay their frustration in filling jobs that, although in the free market, may not reflect their potential and aspirations. Future research should integrate qualitative methods to help clarify the service consumers’ and providers’ expectations, experiences, and perceptions more deeply.

Of note, an additional finding is that the only objective variable related to satisfaction with vocational status was education: participants with higher education reported lower satisfaction. This may stem from a perceived gap between their qualifications and the roles targeted in vocational services (46, 47). A recent study highlighted that many positions obtained through vocational services tend to be low-skilled, which may not fully align with the qualifications of individuals with advanced education. Moreover, consumers’ families and friends believed the tasks performed by these participants were below their skill levels, which may have further exacerbated dissatisfaction (23). This result might carry significant practical implications, suggesting that individuals with higher education may face challenges in fully realizing their potential in the vocational services. It might highlight the need for more tailored vocational services that align with their skills and aspirations.

Significant correlations between perceived skills/perceived support and satisfaction with vocational status were found among people with SMI who consume vocational services in Israel—regardless of the nature of the service consumed. Although this study was cross-sectional and the findings need further corroboration, it used a broad national sample from vocational services throughout the country. Our findings emphasize the importance of the subjective experience of perceived skills and perceived support, identifying client needs to “skill-up,” and tailoring services accordingly. Emphasis should be placed on providing the skills and support consumers need to feel satisfied in the work domain, even when it is more rehabilitative in nature, and on engaging them in meaningful activities without a formal employer–employee relationship. Nevertheless, given the importance of consumers recognizing their skills and abilities, the central role of perceived skills and support and the role of services to support and enhance that process should not be overlooked. In the context of supported employment, our findings reinforce the importance of person–job fit (26) and tailored support to promote satisfaction with vocational status. Hence, the role of vocational services should primarily be to match consumers’ preferences and skills with the offered position.

Our findings should be considered in light of the somewhat narrow operationalization of “job satisfaction” in a one-question measurement of this construct. Full understanding of the construct requires a comprehensive approach that encompasses additional aspects, such as personal fulfillment, engagement in meaningful activities, occupational self-efficacy, and workplace accommodations, which are vital to job satisfaction (22, 23, 30). However, despite its narrow perspective, our study is, to our knowledge, the first to examine differences in vocational status satisfaction between prevocational services and supported employment and their predictors.

Future research should explore satisfaction longitudinally and focus on consumers who transitioned from prevocational services to supported employment. While the significantly higher vocational satisfaction reported by the SE group in our study may stem from their engagement in competitive employment, which aligns more closely with research indicating that competitive employment provides intrinsic and extrinsic rewards that contribute to greater satisfaction, we acknowledge that additional factors, such as person–job fit and individualized support, may also play an important role.

Future investigation of the mechanisms underlying the unique contributions of perceived vocational skills and support is also needed. For example, studies could examine which vocational skills (social or job-specific) seem more influential and the role of low-skill activities in the perceived vocational skills in the different services.

By focusing on perceived support and skills acquisition, organizations can significantly improve satisfaction with vocational status among individuals with SMI, which promotes recovery and well-being at the personal level and contributes to the national workforce at the societal level.

## Data Availability

The datasets analyzed for this study are protected under the IRB regulations. Specific data requests can be made to the corresponding author.

## Footnotes

1 We included participants older than the normal retirement age because we define *vocational activities* to include activities outside formal employment (e.g., participation at vocational support centers or sheltered workshops). These activities often play a critical role in recovery and quality of life for individuals with SMI, irrespective of age. They allow people with SMI to perform meaningful occupations that contribute significantly to their perceived skills, support, and overall satisfaction with their vocational status, which are key aspects of our study.
